# Prayer

**Published:** 1890-06-28

**Authors:** 


### Words of Consolation.

### PRAYER.

When in good health people imagine that prayer will be their chief consolation in illness, but unfortunately, when the time of sickness comes, they often find prayer a subject of great trouble. It seems to them that it should be simply a blessing, but mind and body are so closely connected that the sufferings of the body prevent the mind from exercising itself freely. "The spirit indeed is willing, but the flesh is weak."

You seem to have so much to ask, so much to pray for, that when you try to fix your mind in prayer a stupor comes over you, and in vain you strive to rouse yourself. In the end you start up with a feeling of horror at having fallen asleep over your prayer, even while speaking to God. Sometimes your thoughts seem fixed—not so much that they wander, but that they are utterly gone. You have no control over them. It is as if your mind were giving way, and you wonder what is coming on you. Fear not; it is only weakness that assails you, and which, if it please God ever to remove, and give you back your wonted strength, will be renewed also. In the meantime do not struggle, but be still. It is the part of faith to believe that, since nothing is of chance, He without whom not a sparrow falleth to the ground has appointed this accident of your life. He knoweth every throb of your brow, each shoot of pain, each hard-drawn breath, each beating of the fevered pulse, each sinking of the aching heart. Try, then, to lie calmly in His hands; this very submission is prayer. Silence and submission are your offerings now, and will be as acceptable in His sight as were your prayers in other days. We cannot tell what God will work, nor how, day by day, you may grow more like the ever-blessed Son, whose pleasure was to submit Himself to the will of His heavenly Father.

Perhaps you are looking for something that has never been yours in health. In past years you have considered prayer a hard and uninteresting duty. Now your heart is changed and softened by illness, and at times you feel so sorry for a life spent in carelessness or sin that you long to pour out your heart before God, tell Him of your repentance, and beg Him to send you the help which will make it truly sincere and lasting. If you could only do that, if you could only realize God's presence! But you cannot, and, what seems worse to you, have no earnest and lively desire to realize it. You are afraid of God, that is your only acute feeling on the subject; afraid to ask Him anything, neither can you frame your wishes into words. But words in such a case are unnecessary. He knows your desire, and your groaning is not hid from Him. He looks at your heart and sees there the things which you long to express but cannot. He knows all, and will hea-, and in due time answer.

